# The CXCR4 Antagonist AMD3465 Regulates Oncogenic Signaling and Invasiveness *In Vitro* and Prevents Breast Cancer Growth and Metastasis *In Vivo*

**DOI:** 10.1371/journal.pone.0058426

**Published:** 2013-03-06

**Authors:** Xiaoyang Ling, Erika Spaeth, Ye Chen, Yuexi Shi, Weiguo Zhang, Wendy Schober, Numsen Hail, Marina Konopleva, Michael Andreeff

**Affiliations:** Department of Leukemia, University of Texas M. D. Anderson Cancer Center, Houston, Texas, United States of America; University of South Alabama, United States of America

## Abstract

CXCR4, the receptor for stromal-derived factor-1, is reportedly involved in breast carcinogenesis. However, the mechanisms through which CXCR4 contributes to breast cancer cell growth and metastases are poorly understood. In this study, we examined the putative *in vitro* and *in vivo* anti-cancer effects of the specific CXCR4 inhibitor AMD3465. Here, we report that AMD3465 triggers a reduction in breast cancer cell invasiveness *in vitro*, and promotes marked changes in oncogenic signaling proteins including a reduction in STAT3, JAK2, AKT, and CXCR4 phosphorylation and the reduced expression of GSK3 and cMYC. Using three breast cancer cell lines as murine syngeneic immunocompetent breast cancer models, we found that AMD3465 inhibited breast tumor formation and reduced tumor cell metastases to the lung and liver. Furthermore, treatment with AMD3465 significantly reduced the infiltration of myeloid CD11b positive cells at the aforementioned metastatic sites as well as the spleen implying this agent could regulate the formation of the tumor microenvironment and conceivably the premetastatic niche. In conclusion, our studies suggest that AMD3465 inhibits breast cancer growth and metastases by acting on tumor cells as well as immune cells that constitute the tumor microenvironment. This process appears to be regulated, at least in part, through the modulation of oncogenic signaling that includes the STAT3 pathway. Thus, CXCR4 could be a novel target for breast cancer therapy.

## Introduction

In breast cancer patients, metastases remain a major cause of disease morbidity and mortality. Breast cancer metastases frequently follow a pattern of dissemination in humans that results in the formation of lesions in the lymph nodes, lungs, liver, and bone marrow [1], [2]. Cross talk between cancer cells and their microenvironment is considered an essential event in tumorigenesis, invasion, and metastasis [1], [3], [4]. Specifically, interactions between transformed epithelial cells and their surrounding stroma may decide the fate of evolving cancers [5], since signals from the microenvironment profoundly influence the survival and migration of cancer cells [6]. Increasing evidence shows that CXCR4 and its ligand stromal-derived factor-1 (SDF-1α, also known as CXCL12) may play a critical role in the organ-selective process of tumorigenesis and metastasis including those observed in breast cancers [7]–[9]. For example, CXCR4 expression in tumor cells has been described to be attendant with oncogenic events such as hypoxia [10], RET/PTC mutations [11], [12], EGFR variant-mediated invasion [13], and HER2 overexpression [14].

CXCR4 expression has been established as a prognostic marker in many cancer cell types including breast carcinomas [8], [15]–[17], and the SDF-1α-CXCR4 signaling axis has been associated with breast cancer metastasis [18], [19]. The SDF-1α-CXCR4 interaction promotes tumor progression by several possible mechanisms [3], [20], [21]. For example, SDF-1α that is secreted by stromal cells acts as a chemoattractant allowing the metastatic spread of tumor cells to various cellular niches, such as bone marrow, and ultimately fosters the growth and survival of these cells [15], [22], [23].

Several novel CXCR4 antagonists have shown promising *in vitro* anti-cancer activity in several tumor cell types, including those derived from breast. Furthermore, using animal tumor models, encouraging results from our group and others have indicated that CXCR4 antagonists have *in vivo* anti-cancer activity as well [11], [24], [25]. However, the mechanistic bases (e.g., the modulation of oncogenic signaling and tumor microenvironment) for these effects merit further investigation [11], [26], [27].

Constitutively activated STAT3 has been documented as a key driver of breast cancer growth and metastasis [14], and we have previously reported that STAT3 knockdown in breast cancer cells diminishes CXCR4 expression and inhibits breast cancer growth and metastases in an *in vivo* tumor transplant model [28], [29]. Therefore, we sought to investigate the reciprocal relationships between CXCR4 and oncogenic mediators like STAT3 as a potential mechanistic underpinning in breast tumorigenesis. Using *in vitro* assessments and syngeneic immunocompetent murine breast cancer models, we here report potential mechanisms through which the small molecule antagonist of CXCR4, AMD3465, can inhibit breast cancer growth and metastasis, and demonstrate the biologically relevant modulation of oncogenic signaling and tumor microenvironment by AMD3465.

## Methods

### Cell Lines, Antibodies, and Reagents

The 4T1, 4T07, and 168Farn cells were kindly provided by Dr. Fred R. Miller (Wayne State University School of Medicine, Detroit MI). These murine breast cancer lines were independently derived from spontaneous breast cancers originating in BALB/c mice [30]. Firefly luciferase–tagged 4T1 cells (ffLuc-4T1) were produced as described previously [28]. 4T07 and 168Farn cells were tagged with luciferase and green fluorescent protein (GPF), respectively via lentiviral infection as described previously [29]. The cells were maintained in Dulbecco’s modified Eagle medium (DMEM) supplemented with 10% fetal bovine serum (purchased from Invitrogen Corporation, Carlsbad, CA).

Anti–pTyr-STAT3 (pTyr-705), STAT3, pAKT (pSer 473), AKT, cMYC, JAK2, pJAK2, GSK3, pERK1/2, PTEN and MMP2 antibodies were purchased from Cell Signaling (Beverly, MA). The anti CD11b antibody was purchased from Abcam (Cambridge, MA), and the anti–β-actin from Sigma Life Science (St. Louis, MO). A cell invasion kit was purchased from Chemicon (Temecula, CA). D-Luciferin for firefly luciferase was purchased from Caliper LifeScience (Hopkinton, MA) and the anti-pCXCR4 (S339) and anti-green fluorescent protein (GFP) antibodies (ab38689) were purchased from Abcam (Cambridge, MA). AMD3465 was kindly provided by Genzyme Corporation (Cambridge, MA).

### Animals

Female BALB/c mice (8 wk old) were purchased from Charles River Laboratories (Wilmington, MA) and maintained at the M. D. Anderson Cancer Center animal facility. The experiments were conducted under a protocol approved by the Institutional Animal Care and Use Committee (IACUC) of the M. D. Anderson Cancer Center.

### Western Blotting

Western blotting was performed as previously described [29]. In brief, the cells were treated with AMD3465 or phosphate-buffered saline (PBS, control), trypsinized, and centrifuged for 5 min at 300×g at 4°C. The cell pellets were re-suspended with lysis buffer (Cell Signaling Technology, Boston, MA) for 30 min on ice. The supernatant was collected via centrifugation at 14,000×g for 15 min at 4C°, and the protein concentration was quantitated for SDS-PAGE and Western blotting. The proteins characterized by Western blotting were separated using precast gels (Bio-Rad, Hercules, CA). Roughly 50 µg of total protein was loaded for each lane. The immunoblots were subjected to densitometric analysis using ImageJ software (National Institutes of Health, Bethesda, MD). The band intensities of the indicated proteins were normalized as a percent of the loading control β-actin.

### Cell Proliferation Assay

4T1 cells were seeded onto 6-well plates at a concentration of ∼5×10^5^ cells per well for triplicate assessments. The AMD3465 concentration that was examined in this assay was 5 µM. The total cell number and cell viability in each well was determined using an automated cell analyzer (Vi-Cell; Beckman Coulter, Miami, FL).

### Apoptosis and Cell Cycle Analysis

The externalization of cell membrane phosphatidylserine was analyzed by the annexin V-based technique as described previously [31] using a kit purchased from BD Biosciences (San Jose, CA). The cell cycle analysis was performed as previously described [29]. Briefly, the cells were fixed with 70% ice-cold ethanol and stained with propidium iodide (PI) solution (i.e., 25 µg/ml PI, 180 U/ml RNase, 0.1% Triton X-100, and 30 mg/ml polyethylene glycol in 4 mM citrate buffer, pH 7.8; all purchased from Sigma Chemical Co., St. Louis, MO). The cellular DNA content (i.e., PI fluorescence intensity) was determined using a FACS flow cytometer (Becton Dickinson, San Jose, CA). The PI histograms were analyzed using ModFit LT software (Verity Software House, Topsham, ME).

### Matrix Gel Invasion Assay

The matrix gel invasion assay was conducted in matrix chambers according to the manufacturer’s (Chemicon Internationals, Billerica, MA) instructions, and has been described in detail in our previous work [29].

### Mouse Tumor Assay

For the *in vivo* tumor studies, ∼7×10^3^ 4T1 cells were injected into the mammary fat pads of 8-wk-old female BALB/c mice. AMD3465 treatment was administered hourly by utilizing a subcutaneous osmotic pump (ALZET, Cupertino, CA). Each pump was loaded with 3 mg of AMD3465 dissolved in 100 µl of PBS to yield an AMD3465 delivery dose of 30 µg per h according to the specifications provided by manufacturer. The control mice received only PBS through the same type of pump. The pumps were replaced after day 7 of the 14-d treatment period. Tumor formation at the inoculation site was monitored using an *in vivo* bioluminescence imaging BLI system (Xenogen-200; Caliper Corp., Alameda, CA).

### Mouse Tumor Metastasis Assay

Three breast cancer cell lines were used in this study in a syngeneic immunocompetent mouse breast cancer model. For the 4T1 model, 7×10^3^ cells were inoculated into mammary gland fat pad as described previously [30]. For the 4T07 and 168Farn models, 1×10^5^ 4T07/luciferase/GFP or 168Farn/luciferase/GFP cells were inoculated into mice mammary fat pads. The AMD3465 treatment in the 4T1 model was administered by osmotic pumps starting 3 d after 4T1 cell inoculation because these cells have a greater metastatic capacity [30]. In 4T07 and 168Farn models, the treatment was started 2 d after resection of the primary tumors. The AMD3465 treatment was given for 14 d. The tumors were resected during this treatment period once they reached 2 to 3 mm in diameter. For the 4T1 cells, it required roughly 35 d to establish these tumors, and this process took between 45 and 50 d for the 4T07 and 168Farn cells. The tumor volume was assessed via surgical calipers. Following treatment and tumor resection, the mice were observed until tumor recurrence, and they were sacrificed when the recurring tumor reached a diameter of 2.5 cm.

Tissue samples were collected at the days indicated, and fixed in 10% buffered formalin. In the 4T1 model, metastatic nodules were counted on hematoxylin and eosin (H&E, Sigma) stained slides (3 slides per tissue sample) from lung and liver. For 4T07 and 168Farn models, nodules were counted with anti-GFP antibody stained slides (3 slides per tissue sample, 4 treated mice and 5 control mice for the 4T07 model, 4 control mice and 4 treated mice for the 168Fran model). All areas of the sections were reviewed. All animal procedures, including anesthesia and euthanasia, were carried out according to an animal protocol that was approved by the IACUC of the M. D. Anderson Cancer Center.

### Imaging

Mice were injected intraperitoneally with 4 mg of D-luciferin suspended in 100 µl of PBS and then imaged within 30 min using a Xenogen-200 *in vivo* BLI system. BLI based on detection of light emission was measured in photons/sec and has been described in detail elsewhere [29].

### Histological Analysis

For histological analysis, organs from mice treated with AMD3465 or PBS (control) were excised and fixed in 10% neutral buffered formalin, embedded in paraffin, sectioned, and stained with H&E. pERK1/2 immunostaining was performed in paraffin-embedded tumor sections, and pAKT immunostaining in tumor sections. The secondary antibody was diluted according the company’s instruction. GFP-positive tumor cells were detected by anti-GFP antibody. The histology slides were analyzed using an Olympus BX 41 microscope equipped with a digital camera (Olympus, Melville, NY).

To subtract tissue auto-fluorescence and enumerate Alexa488 positive and CD11b positive cells within the lung, liver, and spleen sections, we obtained spectral images using Nuance software and an Olympus I×81 microscope with a CRi attachment (CRi; Caliper Lifesciences, Hopkinton, MA). We then quantitatively analyzed CD11b positive cells within the tissue sections by designing an algorithm using InForm Software (CRi; Caliper Lifesciences, Hopkinton, MA) to calculate the percentage of CD11b positive cells in each image based on nuclear 4′, 6′ diamino-2-phenylindole dihydrochloride (DAPI, Sigma) staining. Approximately 2000 cells were analyzed per field of view at a magnification of 20X. Ten fields per slide were assessed, and three slides constituted a sample. In addition, CXCR4 and CD11b double fluorescent staining was carried to determine the CD11b positive cells that also expressed CXCR4. The percentage of CD11b and CXCR4 double positive cells in the tissue samples was determined by InForm software (CRi, Wobum, MA).

### Statistical Analyses

The statistical significance between the means of two groups or more was determined using a two-sided, unpaired *t* test or a one-way ANOVA with Dunnett’s post test, respectively (GraphPad Prism version 6.0 software, GraphPad Software, Inc., San Diego, CA). Where indicated, the results are expressed as the mean value of triplicate samples ± SD (error bars). All means ± S.D. for triplicate samples were calculated with Microsoft Excel 2003 SP2 software (Microsoft Corporation, Seattle, WA). In all statistical analyses, the results were considered significant for p<0.05.

## Results and Discussion

### AMD3465 does not Induce Apoptosis or Block the Proliferation of 4T1 Cells *In Vitro*

We [25], [32] and others [33] have used low micromolar (e.g., ≤10 µM), physiologically relevant concentrations of AMD3465 as a CXCR4 inhibitor apparently with no obvious off-target effects *in vitro*. A recent study showed both a decrease in cell proliferation and the induction of apoptosis in tumor cells by CXCR4 inhibition with AMD3465 [11]. To determine if this agent, and potentially the inhibition of CXCR4, was cytotoxic or cytostatic in 4T1 cells, we exposed these cells to 1, 2.5, 5, and 10 µM AMD3465 concentrations for 24 and 48 h. Doing so, we observed no discernible induction of apoptosis as assessed by annexin V/PI staining (data not shown). Furthermore, these AMD3465 treatments had no effect on cell growth (as detected by changes in cell number) or cell cycle distribution (as detected by PI DNA staining) following the same exposures (data not shown). While we cannot exclude the possibility of off-target effects in the 4T1 cells exposed to the aforementioned AMD3465 treatment regime, it was evident that low micromolar concentrations of this agent were insufficient to trigger cytostasis and/or apoptosis in these cells.

### AMD3465 Inhibits 4T1 Cells Invasiveness and Modulates Oncogenic Mediators *In Vitro*

**Figure 1:**
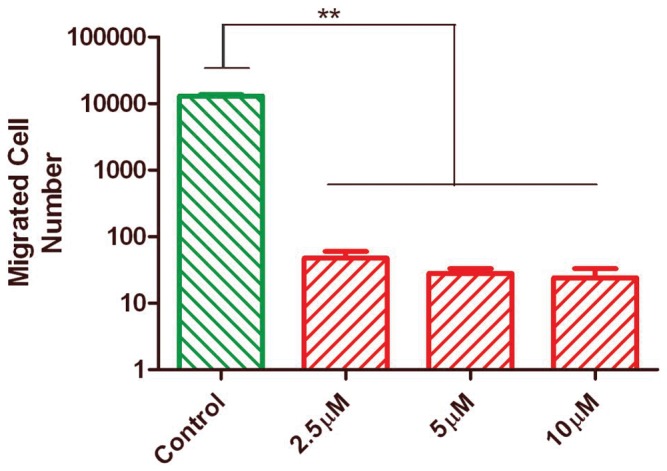
AMD3465 affects the *in vitro* invasiveness of 4T1 cells. 4T1 cells in serum-free medium were seeded in matrigel transwells and allowed to migrate 48 h towards compartments with medium containing 10% FBS without (PBS) and with AMD3465 (2.5, 5, and 10 µM) present. All of the samples were conducted in triplicate and expressed as the mean value ± SD (error bars, **p<0.001).

It has been reported that 4T1 cells are highly metastatic, which can be reflected by their invasive behavior *in vitro* [30]. To determine whether AMD3465 could possibly impede the migration of these cells *in vitro*, we utilized a matrigel invasion assay. Exposing the 4T1 cells to 2.5, 5, or 10 µM AMD3465 for 48 h significantly inhibited their invasiveness compared to the PBS-treated control cells (Fig. 1), implying that CXCR4 inhibition was potentially associated with this process. The ability of this agent to inhibit cell invasiveness without triggering a cytostatic or apoptotic effect in the 4T1 cells would suggest that a subtle AMD3465-induced modulation of oncogenic signaling had occurred in these cells that required additional characterization.

Our group [32] and another [33] have shown that AMD3465 blocks the interaction between CXCR4 and SDF-1α, which results in the dephosphorylation (i.e., inactivation) of CXCR4. However, the signaling mechanisms and related effects of AMD3465 on breast cancer cells have not been extensively elucidated *in vitro*. We therefore investigated effects of the AMD3465 treatment on the expression levels of several kinases, which are known to be associated with breast cancer development, in the 4T1 cells.

**Figure 2:**
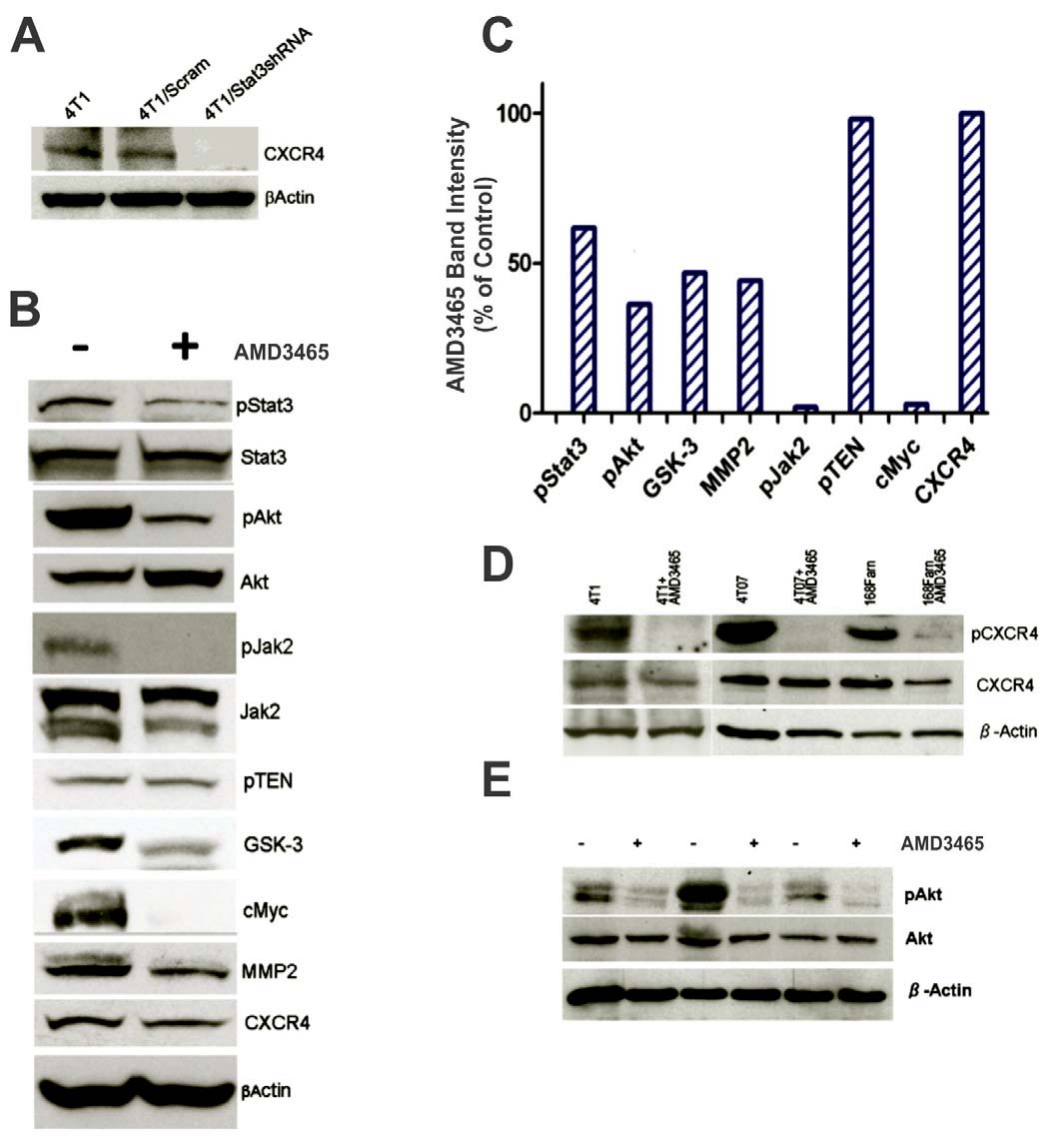
AMD3465 modulates intracellular oncogenic signaling mediators in mouse breast cancer cell lines. **A,** A western blot analysis showing a 24-h knockdown of STAT3 expression using shRNA in the 4T1 cells and the concomitant abrogation of CXCR4 expression. **B,** Western blot analysis of oncogenic intermediates following a 24-h treatment of the 4T1 cells with 5 µM AMD3465. The band intensities ware normalized relative to β-actin expression and presented as % of Control in **C**. **D** and **E,** Western blot analyses of oncogenic intermediates following a 24-h treatment of the 4T1, 4T07, and 168Fran cells with 5 µM AMD3465.

Constitutively activated STAT3 has been found in 4T1, 4T07, and 168Farn cells [28]. Therefore, we wanted to determine if the modulation of STAT3 levels via RNA interference could also regulate CXCR4 expression as a potential indicator of cooperatively between these signaling proteins. Interestingly, when STAT3 was knocked down in the 4T1 cells via this method the expression of CXCR4 was also completely abrogated (Fig. 2A). This would suggest that the expression of CXCR4 was associated with a possible negative feedback loop that was controlled, at least in part, by STAT3.

Next, we examined the effects of AMD3465 on STAT3 and several oncogenic intermediates associated related signaling axes like AKT and JAK in the 4T1 cells. We exposed the 4T1 cells to 5 µM for 24 h. As shown in Fig. 2B, this treatment promoted a reduction of pAKT by approximately 70%. Similarly, JAK2 was down regulated and the expression of pSTAT3, an upstream regulator of AKT, was reduced by approximately 50%. Moreover, the cell proliferation and apoptosis-associated protein cMYC was completely down regulated, the expression of the metastasis-associated protein MMP2 was diminished by approximately 50%, and the expression of the angiogenesis regulator GSK3 was almost completely abolished. In addition, we observed no obvious changes in the protein expression levels of PTEN, AKT, and CXCR4. A graphic representation of these observations is presented in Fig. 2C.

The inactivation (i.e., dephosphorylation) of STAT3 and AKT we observed here mirrored the results reported in our previous study that used the knockdown of STAT3 expression by RNA interference to block the induction of breast tumors in immunocompetent mice [34]. Furthermore, the inactivation of JAK2 appeared to represent a negative feedback effect obtained by inactivation of STAT3 given the observed partial inactivation of STAT3 after the 4T1 cells treated with AMD3465. Indeed, STAT3 inactivation has previously been reported concomitant with SRC inactivation in the 4T1 cells [28].

cMYC is an oncoprotein closely associated with cancer cell proliferation and apoptosis [35]. In our previous reports, either the knockdown of STAT3 [28] or STAT3 inactivation by CCDO-Me [29] resulted in down-regulation of cMYC in 4T1 cells without affecting proliferation. Likewise, we did not detect changes in 4T1 cell proliferation and apoptosis when cMYC expression was down regulated after exposure to AMD3465. This may be an artifact of the *in vitro* culture method, or possibly reflect the subtle the participation of multiple oncogenic signaling networks in these cells.

MMP2 expression is associated with a metastatic breast cancer phenotype, as is the overexpression expression of GSK-3 [36]. Components of the mitogen-activated protein kinase pathway, such as ERK1/2, have been correlated with MMP2 expression in a rat breast cancer brain metastatic model [11], [37]. GSK3 has been shown to function as a nodal point of convergent signaling pathways in endothelial cells to regulate vessel growth; thus, it has been considered integral for tumor angiogenesis [38]. Similarly, it is well established that the activation of AKT triggers GSK3 phosphorylation, and pGSK3 in turn promotes angiogenesis through GSK/catenin signaling. Given that angiogenesis is a critical event for cancer progression, we speculate that the down regulation of GSK3 could be important as an anti-metastasis strategy.

It is has been reported that CXCR4 is not expressed in normal breast tissue [39]. However, the 4T1, 4T07, and 168Farn cells express high levels of this receptor [27], [30]. The effects of AMD3465 on CXCR4 (Fig. 2D) and pAKT (Fig. 2E) expression were similar among the 4T1, 4T07 and 168Farn cells. This would suggest that this agent was functioning by a common inhibitory mechanism in breast cancer cells.

### AMD3465 Modulates Oncogenic Mediators and Inhibits Breast Cancer Growth and Metastasis *In Vivo*

Our *in vitro* investigations thus far demonstrated that oncogenic regulators could be modulated by the AMD3465 treatment of breast cancer cells *in vitro*. Consequently, we wanted to determine whether this agent had similar effects on these cells *in vivo*. Studies using AMD3465 in xenograft brain tumor models showed substantial inhibition of tumor growth [11], [37]. However, given the immunocompromised nature of xenograft models, the biological significance of the role of tumor metastasis and tumor microenvironment in this process could not be assessed fully. Therefore, we opted to use an immunocompetent syngeneic breast cancer model to provide a more clinically relevant system to investigate the effectiveness of CXCR4 blockade by AMD3465 in breast tumor growth and metastasis.

**Figure 3:**
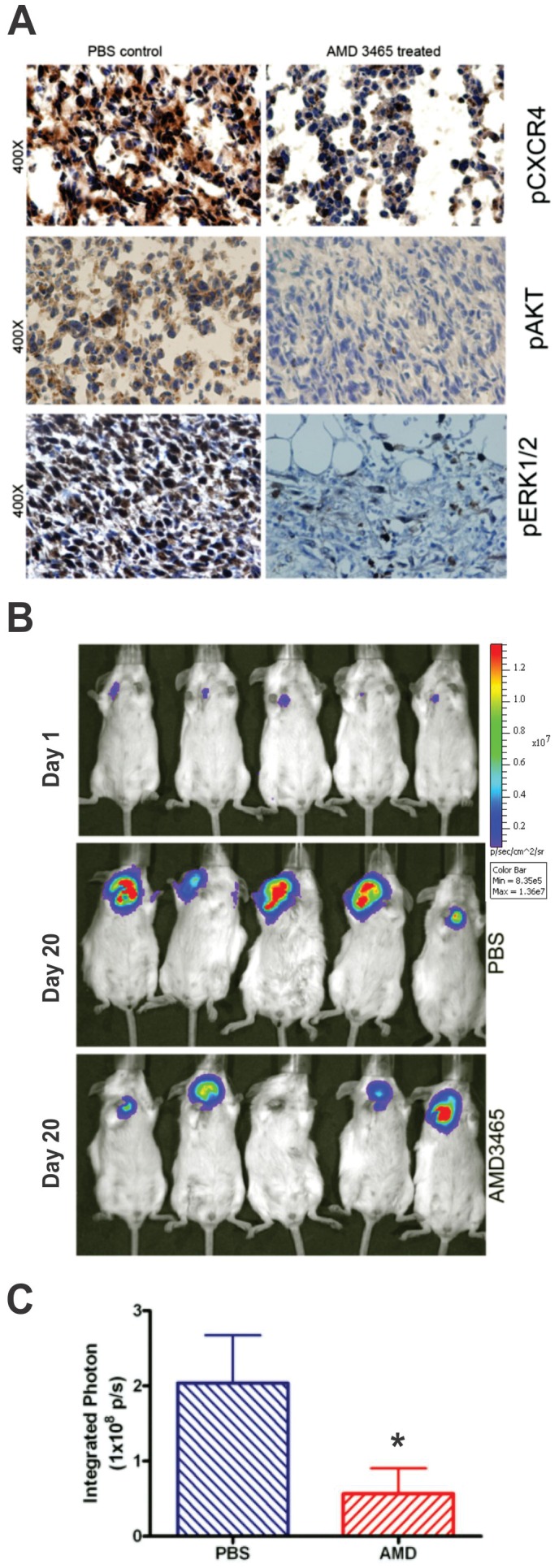
AMD3465 inactivates CXCR4 in 4T1 tumors and slows tumor progression. **A,** Tumor-bearing mice were injected with AMD3465 a single subcutaneous dose of 2.5 mg/kg. The tumor tissue was collected 1 h after treatment and sectioning was carried out. Immunohistochemical staining of pCXCR4, pAKT and pERK1/2 positive tumor cells can be seen in PBS controls compared to AMD3465 treated tumor sections. The slides were analyzed with an Olympus BX 41 microscope equipped with a digital camera (Olympus DP70). **B,** The top panel illustrates the BLI of 5 representative mice 1 d after injection of the 4T1 cells. The middle panel shows representative BLI in 5 mice treated with PBS 20 d after tumor injection, and the lower panel displays imaging of the 4T1 tumor masses following a similar exposure to AMD3465 (please see the Methods section for details). **C,** A bar graph representation of the end-point integrated photon (photons/cm^2^/sec) data collected in the experiment described in **B**. The tumor size was measured by BLI between control mice (n = 5) and AMD3465-treated mice (n = 10) and are expressed as the mean value ± SD (*p<0.05).

It is known that 4T1 cells have a constitutively activated STAT3, which drives tumorigenesis [34]. Moreover, as we mentioned previously, CXCR4 has been shown to activate STAT3 [28]. Thus, we speculated that 4T1 tumor formation *in vivo* should be affected by the CXCR4 inhibitor AMD3465. In order to assess this likelihood, we examined the short-term effect of AMD3465 of oncogenic signaling molecules *in vivo*. We performed immunohistochemical staining of 4T1 tumor samples from mice treated with either AMD3465 or PBS (control) for 1 h. We found that the phosphorylation status of CXCR4, AKT, and ERK were diminished considerably in the tumor samples from the AMD3465-treated mice compared to similar samples from the PBS-treated control animals (Fig. 3A). Furthermore, a long-term exposure to this agent markedly reduced primary 4T1 tumor formation in mice (Fig. 3 B and C). Given the tissue protein changes observed following the short-term exposure to AMD3465 (Fig. 3A) and the ability of this agent to retard long-term tumor formation in mice (Fig. 3 B and C), we surmise that the inhibition oncogenic signaling via CXCR4 blockade is indeed important for breast tumor formation.

**Figure 4:**
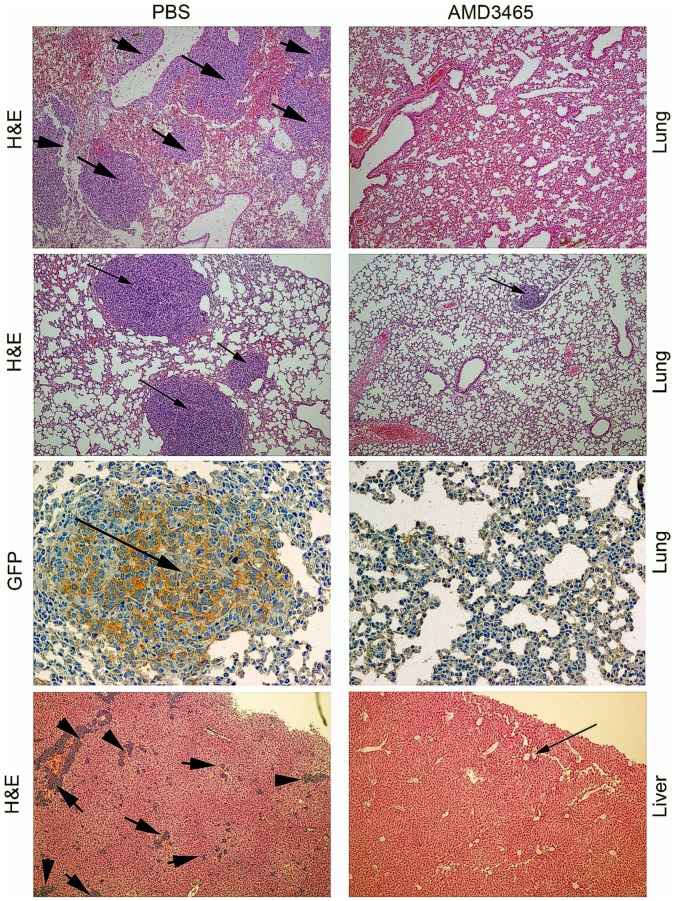
AMD3465 reduces tumor metastases in a syngenic breast cancer model. The size/number (indicated by arrows) of metastatic nodules in the 4T1 tumor bearing mice treated with PBS (control) or AMD3465 in both the lung and liver as determined by H&E staining. We also confirmed the metastatic nodules were GFP positive as were the primary tumors. A detailed treatment procedure for the metastasis assay is described in the Method section.

To evaluate the possible effect of AMD3465 on breast cancer metastasis, we simulated a clinical situation consisting of an AMD3465 treatment prior to tumor resectioning (please see the Methods section). Using this scenario in the immunocompetent syngeneic breast cancer model, we observed a statistically significant decrease in mouse pulmonary and liver metastatic nodules by H&E staining and by staining for the presence of GFP positive tumor cells in the metastatic lesions in the 4T1, 4T07, and 168Farn cells (Table 1). Representative histology sections illustrating these effects in the 4T1 mouse model are presented in Fig. 4. While breast cancer metastases typically form also in the bone marrow and lymph nodes in humans [1], [2], we examined only the lung and liver tissues in this mouse tumor model because these two sites reportedly accumulate the highest number of lesions [30].

**Table 1:** AMD3465 Inhibits Breast Cancer Metastases.

4T1 Cell Model					
PBS Group			AMD3465 Group		
Animal #	Lung Met.	Liver Met.	Animal #	Lung Met.	Liver Met.
1	22	110	1	0	0
2	41	89	2	0	0
3	17	36	3	0	0
4	19	26	4	1	0
5	15	27	5	2	20
6	10	30	6	0	0
7	20	46	7	0	0
			8	2	1
			9	5	0
Mean +/− S.D.	20.8+/−9.8	52+/−33.7	Mean +/− S.D.	1.1+/−1.7*	2.3+/−6.6*
**4T07 Cell Model**					
**PBS Group**			**AMD3465 Group**		
**Animal #**	**Lung Met.**		**Animal #**	**Lung Met.**	
1	3		1	0	
2	5		2	1	
3	6		3	2	
4	8		4	1	
5	6				
Mean +/− S.D.	5.6+/−1.8		Mean +/− S.D.	1.0+/−1**	
**168Farn Cell Model**					
**PBS Group**			**AMD3465 Group**		
**Animal #**	**Lung Met.**		**Animal #**	**Lung Met.**	
1	4		1	0	
			2	1	
3	6				
4	5		4	3	
5	4		5	1	
Mean +/− S.D.	4.8+/−1		Mean +/− S.D.	1.3+/−0.9**	

Site and number of metastases (Met.),

* p<0.001 and **p<0.01 statically significant differences.

Interestingly, in the AMD3465 treated mice we observed a decrease in the formation of blood vessels in the tumor tissues (data not shown). GSK3 was dramatically down regulated after the 4T1 cells were treated with AMD3465 *in vitro* (Fig. 2A), and we observed decreased angiogenesis as indicated by the diminished presence of intratumoral blood vessels in 4T1 tumors relative to the PBS-treated mice (data not shown). Thus, the observed inhibition of tumor cell growth by AMD3465 in this mouse model could have been achieved, at least in part, via the GSK3 inactivation and decreased angiogenesis. Together, these data strongly suggest that the use of a CXCR4 inhibitor like AMD3465 as a preoperative therapy could be useful in patients with potentially metastatic breast cancers.

### AMD3465 Treatment Diminishes Metastatic Site CD11b Positive Cells

**Figure 5:**
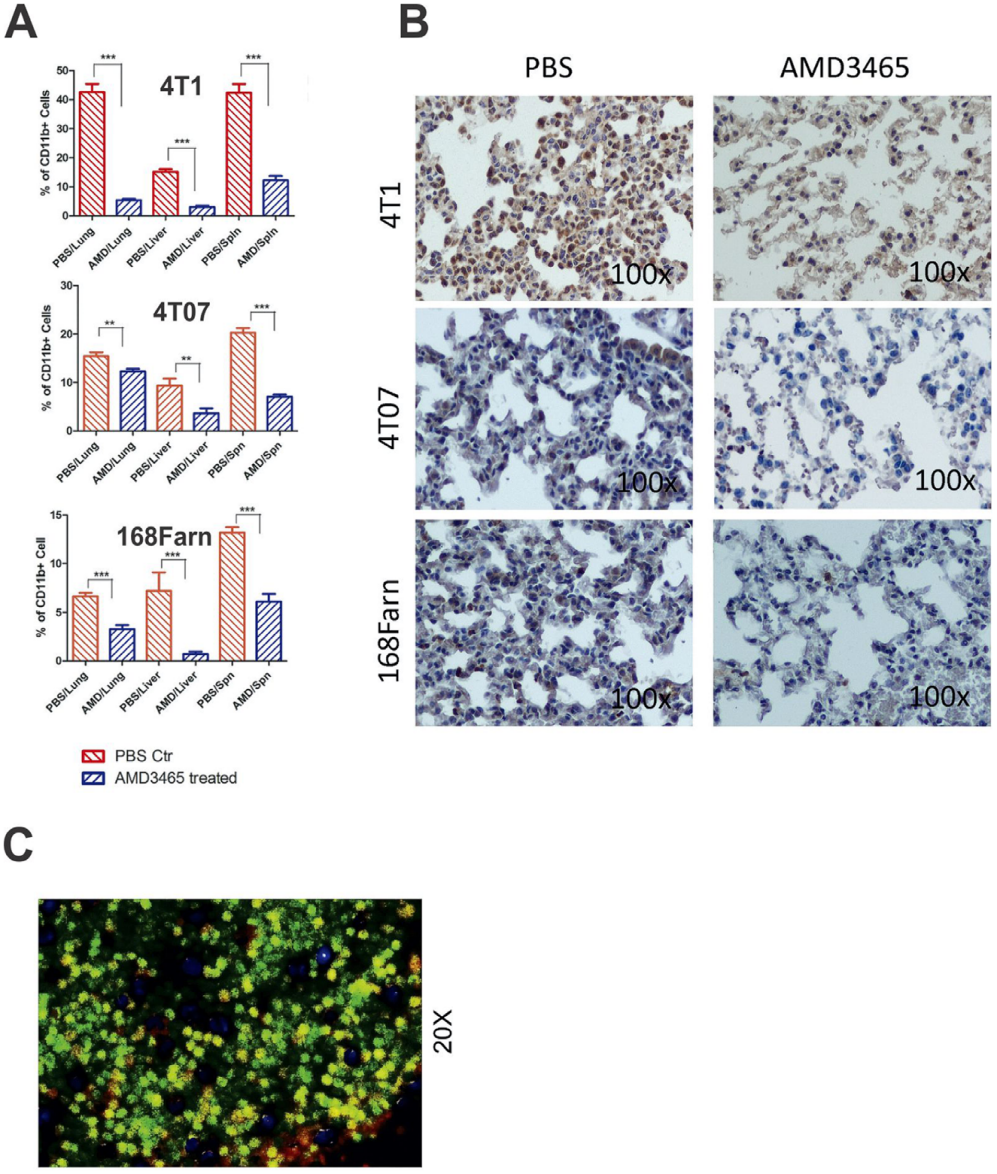
AMD3465 reduces CD11b positive cells within metastatic lesions . **A,** A quantitative representations of CD11b positive cells in the lungs, liver, and spleen of immunocompetent syngeneic mouse model with the indicated cell line were calculated and the results are shown as bar graphs. The reductions in CD11b positive cells after a 14-d AMD3465 treatment in lung, spleen, and liver was observed in all three different breast cancer cell lines in an immunocompetent syngeneic mouse model. The percent positive cells were calculated based on total number of cells counted per image in triplicate samples, and expressed as the mean value ± SD (error bars) (**p<0.01 and ***P<0.001). **B,** A qualitative immunohistochemical depiction of the CD11b positive cells in representative lung tissues is shown for the 4T1, 4T07, and 168Farn cells in the immunocompetent syngeneic mouse model. **C,** Co-staining of metastatic nodules with CXCR4 (green fluorescence) and CD11b (red fluorescence) revealed ∼66% of the cells in the field are positive both for CD11b and CXCR4 (resulting yellow fluorescence) based on quantitative analysis of 10 images of the spleen tissue harvested from PBS-treated mice.

Increasing evidence advocates that tumor-associated macrophages can contribute to cancer cell growth and survival, and may influence the formation of a premetastatic niche for populations of circulating tumor cells [40]. CD11b positive cells have been defined as a myeloid-derived cell lineage, and they share the same phenotype as macrophages. In this study, there were noticeably fewer CD11b positive cells present in lung, liver, and spleen after AMD3465 treatment relative to the respective control tissues for all three cell lines. This decrease was most obvious in the 4T1 model versus the 4T07 or 168Fran models (Fig. 5A).

The CD11b positive myeloid cells appeared to form small clusters within the breast tumors (Fig. 5B and C), demonstrating a role for these cells contributing to the tumor cell microenvironment [41]. The majority (i.e., roughly 66%) of the CD11b positive cells detected in spleen tissue co-expressed CXCR4 in the control mice (Fig. 5C). This would suggest that the AMD3465 treatment affected both the tumor cells and the immune CD11b positive cells involved in the formation of the tumor microenvironment. CXCR4 has been reported to be expressed on CD11b positive cells [42], which constitute a subset of the macrophages that have been demonstrated to promote tumor growth [12]. Given our observations and fact that the presence of CD11b positive cell has been suggested to be associated with the premetastatic niche [22], we speculate that the AMD3465-induced reduction of the CD11b positive cell population may have contributed to the inhibition of metastases.

### Conclusions

In summary, AMD3465 inhibits breast cancer cell invasiveness *in vitr*o, and oncogenic signaling both *in vitro* and *in vivo*. The later observations corresponded with the ability of this agent to decrease the growth of breast cancer cells, as well as the metastatic potential of these cells, in an immunocompetent syngeneic mouse model. Together, these results strongly suggest that CXCR4-inhibition can effectively block breast cancer cell dissemination, at least in part, by modulating oncogenic mediators like MMP2, GSK3, cMYC, AKT and STAT3. Finally, we further propose that AMD3465 treatment can inhibit the metastatic potential of the tumor cells by also inhibiting the formation of a nurturing microenvironment like the premetastatic niche, which is seemingly mediated by the infiltration of CD11b positive macrophages.
